# *Delirium* severity and outcomes of critically ill COVID-19 patients

**DOI:** 10.5935/2965-2774.20230170-en

**Published:** 2023

**Authors:** Luciana Leal do Rego, Jorge Ibrain Figueira Salluh, Vicente Cés de Souza-Dantas, José Roberto Lapa e Silva, Pedro Póvoa, Rodrigo Bernardo Serafim

**Affiliations:** 1 Instituto D’Or de Pesquisa e Ensino - Rio de Janeiro (RJ), Brazil; 2 Postgraduate Program in Clinical Medicine, Universidade Federal do Rio de Janeiro - Rio de Janeiro (RJ), Brazil; 3 Polivalente Intensive Care Unit, Hospital de São Francisco Xavier, Centro Hospitalar de Lisboa Ocidental - Lisboa, Portugal

**Keywords:** Delirium, COVID-19, Coronavirus infections, Critical illness, Psychiatric status rating scales, Surveys and questionnaires, Risk factors, Prognosis, Critical care outcomes, Intensive care units

## Abstract

**Objective:**

To investigate the impact of *delirium* severity in critically ill COVID-19 patients and its association with outcomes.

**Methods:**

This prospective cohort study was performed in two tertiary intensive care units in Rio de Janeiro, Brazil. COVID-19 patients were evaluated daily during the first 7 days of intensive care unit stay using the Richmond Agitation Sedation Scale, Confusion Assessment Method for Intensive Care Unit (CAM-ICU) and Confusion Method Assessment for Intensive Care Unit-7 (CAM-ICU-7). *Delirium* severity was correlated with outcomes and one-year mortality.

**Results:**

Among the 277 COVID-19 patients included, *delirium* occurred in 101 (36.5%) during the first 7 days of intensive care unit stay, and it was associated with a higher length of intensive care unit stay in days (IQR 13 [7 - 25] *versus* 6 [4 - 12]; p < 0.001), higher hospital mortality (25.74% *versus* 5.11%; p < 0.001) and additional higher one-year mortality (5.3% *versus* 0.6%, p < 0.001). *Delirium* was classified by CAM-ICU-7 in terms of severity, and higher scores were associated with higher in-hospital mortality (17.86% *versus* 34.38% *versus* 38.46%, 95%CI, p value < 0.001). Severe *delirium* was associated with a higher risk of progression to coma (OR 7.1; 95%CI 1.9 - 31.0; p = 0.005) and to mechanical ventilation (OR 11.09; 95%CI 2.8 - 58.5; p = 0.002) in the multivariate analysis, adjusted by severity and frailty.

**Conclusion:**

In patients admitted with COVID-19 in the intensive care unit, *delirium* was an independent risk factor for the worst prognosis, including mortality. The *delirium* severity assessed by the CAM-ICU-7 during the first week in the intensive care unit was associated with poor outcomes, including progression to coma and to mechanical ventilation.

## INTRODUCTION

In addition to pulmonary manifestations and acute respiratory failure, novel coronavirus disease 2019 (COVID-19) may cause neurological conditions,^(1)^ including encephalopathy, *delirium*, and coma.^(2-5)^ A direct effect of the virus on the central nervous system, the release of inflammatory cytokines, and the activation of the coagulation cascade are some of the underlying mechanisms for neurological complications of COVID-19.^(6)^ Moreover, critically ill COVID-19 patients are frequently exposed to hypoxemia, deep sedation, systemic corticosteroids,^(3,7)^ restrictions on family visits and prolonged mechanical ventilation (VM),^(8)^ which are well-described risk factors for the occurrence of persistent *delirium* in intensive care unit (ICU) patients.^(3,9-11)^ *Delirium* occurrence has a well-documented association with worse patient outcomes, such as increased ICU length of stay (LOS), cognitive decline, depression, postintensive care syndrome and higher short-term mortality.^(12-14)^ Severity and duration of *delirium* are also independently associated with higher mortality and morbidity in the ICU.^(15,16)^

Although there are many studies assessing the incidence and impact of *delirium* in critically ill COVID-19 patients,^(14,17,18)^ few have focused on *delirium* severity in this setting.^(5,13,19)^ Therefore, the aim of the present study is to investigate the impact of *delirium* severity in critically ill COVID-19 patients and its association with the main outcomes.

## METHODS

### Study design and participants

**Figure 1:**
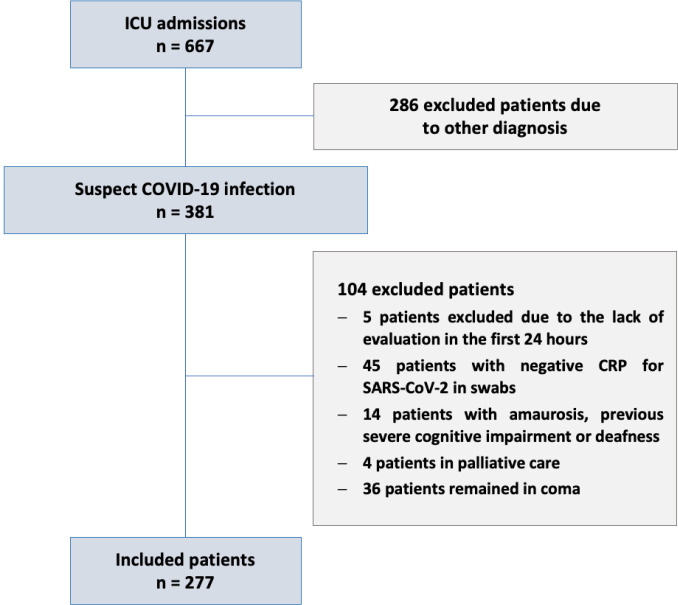
Flowchart of the inclusion of patients.

We conducted a prospective cohort study in the ICUs of two tertiary hospitals in Rio de Janeiro, Brazil, between May 1st and 31st August 2020. All adult patients admitted with clinical and radiological suspicion of COVID-19 were evaluated daily during the first seven days of ICU stay. Subsequently, according to the results of the polymerase chain reaction (PCR) tests, we excluded and removed patients with negative results from the analysis. Only those with a confirmed diagnosis of coronavirus infection by a positive PCR for severe acute respiratory syndrome coronavirus 2 (SARS-CoV-2) in nasopharynx and oropharynx swabs were included in the study. Exclusion criteria were inability to collaborate with the *delirium* assessment (deafness, amaurosis, previous severe dementia or other severe cognitive impairment), persistence of coma (defined by Richmond Agitation-Sedation Scale [RASS] -4 and -5 in first week of admission) and a previous decision of palliative care. Patients who could not be evaluated in the first 24 hours of ICU stay were also excluded. A flowchart of patient inclusion is provided in figure 1.

ICU - intensive care unit; CRP - C-reactive protein; SARS-CoV-2 - severe acute respiratory syndrome coronavirus 2.

The study was approved by the Research Ethics Committee (*Instituto D’Or de Pesquisa e Ensino*, CAAE 17079119.7.0000.5249), with a waiver in the application of informed consent due to the observational nature of the study.

### Data collection

Demographic and clinical data were collected prospectively from the charts, electronic records or patient proxies, including the date of symptom onset and presence of comorbidities (dementia or cognitive deficit, diabetes mellitus, chronic obstructive pulmonary disease, alcoholism, systemic arterial hypertension, heart failure, immunosuppression or active cancer, chronic kidney disease or obesity). The E-predeliric,^(20)^ Charlson Comorbidities Index (CCI),^(21)^ modified Frailty Index (m-FI)^(22)^ and Simplified Acute Physiology Score 3 (SAPS 3)^(23)^ on admission were calculated and recorded. During the first seven days of ICU stay, the RASS,^(24)^ Confusion Assessment Method for the ICU (CAM-ICU)^(25)^ and Confusion Assessment Method for the ICU-7 (CAM-ICU-7, a validated *delirium* severity 7-point scale that graduates and sums each component of the CAM-ICU);^(26)^ the use of systemic corticosteroids, sedatives, and antipsychotics; and laboratory data and Sequential Organ Failures Assessment (SOFA)^(27)^ were checked and recorded daily.

Every morning during the first seven days of ICU stays, a systematic evaluation of sedation, coma and *delirium* was performed by three senior intensivists, most of the time by the same intensivists, using the RASS scale, the CAM-ICU and the CAM-ICU-7,.

Assessment of *delirium* was only possible in patients with RASS scores greater than RASS-3. Patients with RASS-4 and RASS-5 scores were categorized as comas. Patients who had *delirium* on at least one of the days of analysis were considered patients in the group with *delirium*, and patients who did not have *delirium* on any of the days analyzed were considered without *delirium*. Patients who remained in a coma for the entire time of the analysis were excluded. The CAM-ICU-7 mean was calculated by the arithmetic mean of the days that this patient was assessed for *delirium* during the seven days of the analysis.

### Outcomes

Our primary outcomes were *delirium* incidence and severity (measured by the CAM-ICU-7 score) and its association with hospital mortality rates. In addition, we evaluated secondary outcomes: progression to coma and to MV, ICU LOS and one-year mortality in survivors after discharge (these last data were extracted from the governmental database of *Corregedoria Geral do Tribunal de Justiça do Rio de Janeiro* - TJRJ). We emphasize that nonclinical factors had an impact on our length of hospital stay, such as respiratory isolation time and lack of availability of hospital ICU and non-ICU beds to receive these patients.

### Statistical analysis

Data are presented as medians with interquartile ranges (IQRs) for continuous variables and absolute values and percentages for categorical variables. As appropriate, categorical variables were compared using the chi squared or Fisher’s exact test, and continuous variables were compared using the Kruskal-Wallis or Mann-Whitney U test.

*Delirium* severity was described by calculating the CAM-ICU-7 means in the first seven days of ICU stay and stratified accordingly with strata as described in the original article (< 3: mild *delirium*; 3 - 5.99: moderate *delirium* and 6 - 7: severe *delirium*).^(26)^ The length of stay in the ICU and in the hospital was analyzed using the Fine-Gray subdistribution hazard competing risk model.^(28,29)^ The hazard ratio of discharge chance from the hospital and ICU was calculated by comparing *delirium* and non-*delirium* patients with the median time to discharge of the total sample.

The association of *delirium* severity with outcomes was explored using a univariate analysis by estimating the risk ratios. After univariate analysis, variables that presented a p < 0.25 were entered in the multivariate analysis to correlate *delirium* with the primary and secondary outcomes. Multivariable adjusted logistic regression models adjusted by SAPS 3 and frailty were used to estimate the odds ratios (for mortality, late mortality, progression to coma and to MV) and hazard ratios (chance of hospital and ICU discharge) and 95% confidence intervals (95%CI). All tests were two-sided, and statistical significance was defined at a level of 95%CI, with a p value < 0.05. All analyses were performed with R software version 4.2.1 using the final fit and survival packages.

## RESULTS

A total of 277 patients were included in the study (Figure 1), and overall, *delirium* occurred in 101 patients (36.5%). Most patients (70.4%) were men, and the mean CCI was 1.0 (0-3.0). Patients had a mean SAPS 3 score of 47.0 (42.0 - 54.0), and the mean m-FI was 18.2 (9.1 - 27.3).

Patients who presented *delirium* had more comorbidities, were frailer and had higher severity of illness scores (as expressed by a higher CCI, m-FI, SOFA and SAPS 3, respectively), and had higher C-reactive protein - CRP (9.72mg/dL [5.26 - 17.20] *versus* 6.90mg/dL [3.70 - 13.95]; p = 0.048] at admission. The use of sedative and neuromuscular blockage was more frequent in patients with *delirium*: midazolam (37.6% *versus* 15%; p < 0.001), fentanyl (42.5% *versus* 22%; p < 0.001), neuromuscular blockage (9.9% *versus* 7.9%; p = 0.29) and dexamethasone (47.5% *versus* 14.8%, p < 0.001) (Table 1).

**Table 1:** Clinical and demographic variables of the population with and without *delirium*

Clinical characteristics	Non delirium (n = 176)	Delirium (n = 101)	p value
Age	64.5 (52.8 - 72.0)	80.0 (67.0 - 87.0)	< 0.001
Sex (male)	128 (72.7)	67 (66.3)	0.3248
Body mass index	27.69 (24.9 - 31.4)	26.5 (23.8 - 30.5)	0.0848
Alcoholism	4 (2.3)	5 (4.9)	0.2939
Dementia/cognitive impairment	4 (2.3)	22 (21.8)	0.001
Chronic kidney disease	6 (3.4)	4 (3.9)	1.000
Corticosteroid use	56 (31.8)	25 (24.8)	0.2682
Acute respiratory failure	43 (24.4)	59 (58.4)	< 0.001
Hypertension	83 (47.2)	70 (69.3)	< 0.001
Obesity	58 (32.9)	32 (31.7)	0.9329
Imunossupression/cancer	23 (13.1)	24 (23.8)	0.0343
CAM-ICU-7	0 (0.0 - 0.0)	2.50 (1.3 - 4.3)	< 0.001
SAPS 3	44.0 (41.0 - 50.0)	54.0 (49.0 - 57.0)	0.001
Frailty (m-FI)	48 (27.3)	56 (55.6)	0.001
Charlson Comorbidities Index	0 (0.0 - 2.0)	3.0 (1.0 - 6.0)	0.001
E-predeliric	19.0 (14.0 - 26.0)	33.0 (25.0 - 43.0)	0.001
CRP mean Day 1	6.90 (3.7 - 13.9)	9.72 (5.3 - 17.2)	0.0478
Midazolam use	27 (15.0)	38 (37.6)	0.001
Fentanyl use	39 (22)	43 (42.5)	0.001
Neuromuscular blockade use	14 (7.9)	10 (9.9)	0.29
Propofol use	11 (6.2)	19 (18.8)	0.48
Dexmedetomedine use	26 (14.8)	48 (47.5)	0.001
Invasive mechanical ventilation	36 (20.4)	48 (47.5)	0.001

CAM-ICU-7 - Confusion Assessment Method for the Intensive Care Unit-7; SAPS 3 - Simplified Acute Physiology Score 3; m-FI - modified Frailty Index; CRP - C-reactive protein. Results expressed as the median (interquartile range) or n (%).

The in-hospital mortality rates in the *delirium* and non *delirium* groups were 25.74% *versus* 5.11%, respectively (p < 0.001). The additional one-year mortality of patients who were discharged alive from the hospital was 5.3% in *delirium versus* 0.6% in non *delirium* patients, p < 0.001 (Table 2).

**Table 2:** Clinical outcomes of the population with and without *delirium*

Outcomes	Non delirium	Delirium	p value
Length of ICU stay	6 (4 - 12)	13 (7 - 25)	< 0.001
Length of hospital stay	8 (5 - 14)	17 (9 - 37)	< 0.001
Invasive mechanical ventilation days	0 (0 - 0)	2 (0 - 10)	< 0.001
Progression to mechanical ventilation	2 (1.1)	38 (37.6)	< 0.001
Coma	30 (17.0)	44 (43.6)	< 0.001
In-hospital mortality	9 (5.1)	26 (25.7)	< 0.001
One-year (additional) mortality	1 (0.6)	4 (5.3)	< 0.001

ICU - intensive care unit. Results expressed as the median (interquartile range)

Patients were at increased risk of requiring invasive MV after developing *delirium* (OR 51.35 [95%CI 11.65 - 226.35]; p < 0.001) and had a lower chance of discharge from the ICU (HR 0.54 [95%CI 0.40 - 0.71]; p < 0.001)) than those without *delirium*. In multivariate analysis, *delirium* was also independently associated with mortality OR 3.04 (95%CI 1.26 - 7.36); p = 0.014 (Table 3).

**Table 3:** Delirium and outcomes in multivariate analysis

Outcomes	Measures of effect (95%CI)	p value
Chance of hospital discharge	HR 0.5 (0.4 - 0.7)	< 0.001
Chance of ICU discharge	HR 0.53 (0.4 - 0.7)	< 0.001
Progression to mechanical ventilation	OR 51.35 (11.7 - 226.4)	< 0.001
In-hospital mortality	OR 3.04 (1.26 - 7.36)	< 0.014
One year (additional) mortality	OR 1.96 (0.25 - 15.39)	0.521

HR - hazard ratio; OR - odds ratio; 95%CI - 95% confidence interval; ICU - intensive care unit. Results expressed as the median (interquartile range) or n (%).

*Delirium* was classified according to the CAM-ICU-7 mean in three levels of severity: mild, moderate, and severe. A higher level of *delirium* was associated with a higher frailty status prevalence (41.1% *versus* 71.9% *versus* 77%; p < 0.001) and higher in-hospital mortality (17.9% *versus* 34.4% *versus* 38.5%; p < 0.001) (Table 4).

**Table 4:** Clinical scores and patient outcomes relative to the stratification of delirium severity by Confusion Assessment Method for Intensive Care Unit-7

	CAM-ICU-7			p value
	Median (< 3)	Median (3 - 5.99)	Median (6 - 7)	
Total of patients	56	32	13	
Mean SAPS 3	52.0 (44.0 - 57.0)	56.0 (49.0 - 58.5)	54.0 (50.0 - 63.0)	< 0.001
Frailty-m-FI	23 (41.07)	23 (71.88)	10 (76.92)	< 0.001
Mean CAM-ICU-7	1.4 (0.96 - 2.0)	4.0 (3.65 - 5.0)	6.5 (6.0 - 7.0)	< 0.001
ICU length of stay	12.00 (6.00 - 24.25)	16.50 (8.00 - 26.50)	13.00 (5.00 - 25.00)	< 0.001
Hospital length of stay	16.00 (8.00 - 40.00)	17.00 (9.75 - 28.50)	20.00 (13.00 - 44.00)	< 0.001
Evolution to MV	15 (26.79)	13 (40.62)	10 (76.92)	< 0.001
Mortality	10 (17.85)	11 (34.38)	5 (38.46)	< 0.001

CAM-ICU-7 - Confusion Assessment Method for the Intensive Care Unit 7; SAPS 3 - Simplified Acute Physiology Score 3; m-FI - modified Frailty Index; ICU - intensive care unit; MV - mechanical ventilation. The results are expressed as the median (interquartile range) or n (%).

**Figure 2:**
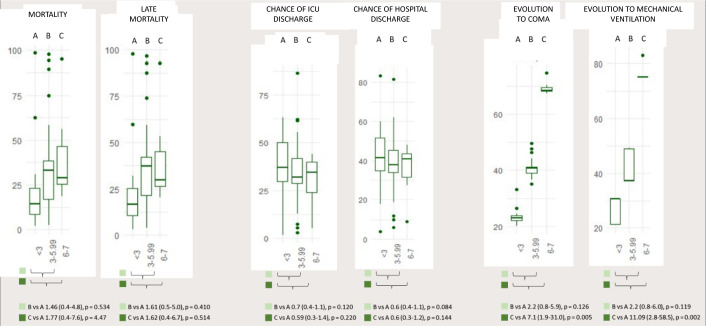
Comparison of the three median strata of *delirium* severity with the following outcomes (in odds ratio or hazard ratio, 95% confidence intervals): mortality, late mortality, chance (in percentage) of intensive care unit discharge, chance (in percentage) of hospital discharge, chance of evolution to coma (in percentage) and chance (in percentage) of evolution to invasive mechanical ventilation. (A) Confusion Assessment Method for the Intensive Care Unit-7 mean < 3; (B) Confusion Assessment Method for the Intensive Care Unit-7 mean between 3 - 5.99; (C) Confusion Assessment Method for the Intensive Care Unit-7 mean > 6..

After multivariate analysis, using mild *delirium* as a reference and adjusting by the SAPS 3 score and frailty, moderate and severe *delirium* had a higher risk of progression to invasive MV (patients who were not in invasive MV and evolved with acute respiratory failure, requiring invasive MV; OR 2.2 [95%CI 0.8 - 6.0]; p = 0.119; and OR 11.09 [95%CI 2.8 - 58.5]; p = 0.002) and a higher risk of progression to coma (OR 2.2 [95%CI 0.8 - 6.0]; p = 0.126 and OR 7.1 [95%CI 1.9 - 31.0 ]; p = 0.005, respectively) (Figure 2). Moderate and severe *delirium*, had comparable results for chance of ICU discharge (HR 0.7 [95%CI 0.4 - 1.1]; p = 0.120 and 0.6 [95%CI 0.3 - 1.4]; p = 0.220) and for mortality (1.46 (95%CI 0.4 - 4.8); p = 0.534 *versus* 1.77 [95%CI 0.4 - 7.6]; p = 0.447), respectively (Table 5).

**Table 5:** Outcomes in *delirium* patients classified by Confusion Assessment Method for Intensive Care Unit-7 mean in multivariate analysis

Outcomes	CAM-ICU-7			
	Mean (3 - 5.99)		Mean (6 - 7)	
	Measures of effect (95%CI)	p value	Measures of effect (95%CI)	p value
Chance of hospital discharge	HR 0.6 (0.4 - 1.1)	0.084	HR 0.6 (0.3 - 1.2)	0.144
Chance of ICU discharge	HR 0.7 (0.4 - 1.1)	0.120	HR 0.59 (0.3 - 1.4)	0.220
Evolution to coma	OR 2.2 (0.8 - 5.9)	0.126	OR 7.1 (1.9 - 31.0)	0.005
Evolution to mechanical ventilation	OR 2.2 (0.8 - 6.0)	0.119	OR 11.09 (2.8 - 58.5)	0.002
Mortality	OR 1.46 (0.4 - 4.8)	0.534	OR 1.77 (0.4 - 7.6)	0.447
Late mortality	OR 1.61 (0.5 - 5.0)	0.410	OR1.62 (0.4 - 6.7)	0.514

CAM-ICU-7- Confusion Assessment Method for the Intensive Care Unit-7; HR - hazard ratio; OR - odds ratio; 95%CI - 95% confidence interval; ICU - intensive care unit.

ICU - intensive care unit.

## DISCUSSION

In our prospective cohort, the incidence of *delirium* was high (36.5%), and it was associated with increased in-hospital mortality, increased ICU and hospital LOS and a higher use of MV, even when adjusted for other severity scores (SAPS 3 and frailty).

The *delirium* occurrence was higher when compared to a similar ICU population without COVID-19.^(15,16,30)^ This finding was similar to that of other studies in critically ill COVID-19 patients.^(31-33)^

Our cohort described a very low one-year mortality of patients with *delirium* after discharge (5.3%); nevertheless, it was 8 times higher than late mortality in non-COVID patients. Despite few studies describing late mortality in patients with COVID-19, our result was similar to other recent observational studies that found only 1% one-year mortality in COVID-19 survivors.^(34)^ Although *delirium* occurrence in non-COVID-19 patients has been associated with a higher 1-year mortality,^(35,36)^ late survival to COVID-19 is closely associated with comorbidities and functional status.^(37)^ In our cohort, *delirium* survivors were less frail (m-FI: 27.3 [95%CI 18.0 - 36.4] *versus* 18.0 [95%CI 9.1 - 27]; p < 0.001), had a lower CCI (CCI: 3.0 [95% CI 2.7-3.2] *versus* 4.8 [95%CI 3.8 - 5.7]; p < 0.001) and had a lower severity score (SAPS3: 50.8 [95%CI 50.4 - 51.3] *versus* 65.1 [95%CI 64.1 - 66.1]; p < 0.001) than nonsurvivors. We also described the correlation between *delirium* severity (according to the mean CAM-ICU-7 assessment) and outcomes.

There are only a few studies comparing *delirium* severity and outcomes in critically ill COVID-19 patients.^(38)^ Only one study described an increase in mortality in coma patients but did not describe subgroups of *delirium* severity.^(37)^ Our study described that the mortality of patients with moderate to severe *delirium* was nearly twice that observed in mild *delirium* (respectively 17.9% and 34.4% *versus* 38.5%; p < 0.001). Similar to rapid reverse *delirium*, mild *delirium* represents a lower risk of death.^(5)^

In our cohort, severe *delirium* was also associated with a high amount of resources used (MV or length of stay). Monitoring *delirium* severity can identify high-risk patients and resource allocation. The imbalance between supply and demand for medical resources during the pandemic highlights the importance of projecting future demands in the ICU regarding *delirium* severity.

*Delirium* diagnosis and monitoring was also a challenge during the COVID-19 pandemic. Spread barriers need to be adopted, and the main emphasis has been placed on organizational barriers in the COVID-19 population. In our sample, E-predeliric had a discriminative performance similar to that of patients without COVID-19, with an issue hindering bedside *delirium* screening. Our performance of the E-predelic score in *delirium* prediction had an area under the ROC curve of 0.783, p < 0.001, which is very similar to that described in non-COVID-19 patients.^(20,38)^ Understanding *delirium* patterns and characteristics can help to select appropriate screening tools and preventive measures for future conditions.

Our study has many strengths, including the prospective design, the bedside data collection (not chart-based method), the size of our sample, the multivariate analysis adjusting for possible confounders and mainly the assessment of *delirium* severity and its prognosis in this population. There are few studies describing outcomes and late mortality in patients with *delirium* and COVID-19, and the CAM-ICU-7 has been underexplored in this population.

While effective pharmacological therapies for *delirium* are not yet available,^(18)^ our data emphasize *delirium* as a predictor of poor outcomes in the ICU population admitted with COVID-19 and the importance of implementing a screening protocol as well as monitoring the severity of *delirium*.

However, some limitations need to be highlighted. First, it is worth noting that our analysis focused only on the first week of ICU stay, which may have underestimated the incidence of *delirium*. However, it is important to recognize that *delirium* is more likely to occur during the initial days of admission.^(5)^ Therefore, early monitoring for *delirium* remains critical in guiding decision-making during ICU treatment. Second, as we performed this study at the beginning of the first wave of the pandemic and there was a large concern about the virus spreading, we limited patient assessment to one visit a day, which may also have reduced our detection of *delirium*. Third, the sample we analyzed may not represent the entire population, as many patients were unable to be evaluated using the CAM-ICU or CAM-ICU-7. This could result in a potentially less severe patient population. A significant number of patients needed MV and deep sedation due to severe hypoxemia. Additionally, many patients require benzodiazepines for sedation due to a shortage of short-acting drugs.^(39,40)^ Fourth, the lack of details regarding corticosteroid use in our study population could represent a confounding bias. It is important to note that our study was conducted in the early stages of the pandemic when there was still controversy and concern about the use of corticosteroids, especially in advanced infection cases. Finally, we evaluated only late mortality, and we did not evaluate other late outcomes, such as the prevalence of functional decline or the presence of posttraumatic stress syndrome.

## CONCLUSION

The incidence of *delirium* was high in COVID-19 patients. *Delirium* was an independent risk factor for the worst prognosis, including mortality during hospitalization, but had a slight impact on 1-year mortality.

Our study emphasizes the applicability of the CAM-ICU-7 scale in the COVID-19 intensive care unit population and reinforces the importance of graduating *delirium* and its correlation with worse outcomes. The *delirium* severity assessed by the CAM-ICU-7 during the first week in the intensive care unit was associated with poor outcomes, including evolution to coma and to mechanical ventilation.
